# Home range size scales to habitat amount and increasing fragmentation in a mobile woodland specialist

**DOI:** 10.1002/ece3.5837

**Published:** 2019-11-18

**Authors:** Riana Gardiner, Kirstin Proft, Sebastien Comte, Menna Jones, Chris N. Johnson

**Affiliations:** ^1^ School of Natural Sciences University of Tasmania Hobart TAS Australia; ^2^ Vertebrate Pest Research Unit NSW Department of Primary Industries Orange NSW Australia

**Keywords:** fragmentation, habitat amount, home range, management, restoration

## Abstract

1. Studies of impacts of fragmentation have focused heavily on measures of species presence or absence in fragments, or species richness in relation to fragmentation, but have often not considered the effects of fragmentation on ranging behavior of individual species. Effective management will benefit from knowledge of the effects of fragmentation on space use by species.
2. We investigated how a woodland specialist, the eastern bettong (*Bettongia gaimardi*), responded to fragmentation in an agricultural landscape, the Midlands region of Tasmania, Australia. We tested whether individual bettongs could adjust home range size to maintain access to essential habitat across three sites differing in degree of fragmentation.
3. We used GPS tracking to measure the home ranges of individual bettongs. Our models tested the effects of habitat aggregation and habitat amount measured at two radii comparable to a typical core range (250 m) and a typical home range (750 m), and habitat quality and sex on individual home range. We also tested the relationship between fragmentation on woodland used to determine whether individuals could compensate for fragmentation.
4. Depending on the spatial scale of fragmentation measured, bettongs altered their movement to meet their habitat requirements. Our top model suggested that at the core range scale, individuals had smaller ranges when habitat is more aggregated. The second model showed support for habitat amount at the core range, suggesting individuals can occupy larger areas when there is a higher amount of habitat, regardless of configuration.
5. Species that are relatively mobile may be able to compensate for the effects of habitat fragmentation by altering their movement. We highlight that any patch size is of value within a home range and management efforts should focus on maintaining sufficient habitat especially at the core range scale.

## Graphical abstract

For a mobile species that can cross gaps, the configuration of patches does not hinder movement given there is sufficient habitat.

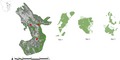

## 1 INTRODUCTION

Habitat loss is a global threat to biodiversity and a challenge for conservation managers (Haddad et al., 2015; Hanski, 2015). The conversion of continuous habitat into smaller and often isolated patches can constrain species distributions and threaten population viability by reducing local population size (Fahrig, 2017). As habitat fragmentation becomes more widespread (Haddad et al., 2015; Lindenmayer & Fischer, 2007; Tilman et al., 2017), management of the habitat that remains in fragmented landscapes will be increasingly important. Such management should be grounded in a detailed understanding of how species respond to the loss and fragmentation of their habitat.

The impacts of habitat fragmentation on wildlife species have generally been described in terms of how landscape configuration influences species richness in fragments or the occupation of fragments by particular species. Effects of fragmentation on these variables are often interpreted in relation to classic metapopulation and island biogeography theories, or more recent ideas such as the habitat continuum and habitat amount hypotheses (Fahrig, 2013; Fischer, Lindenmayer, & Kaitala, 2006; Hanski, 2015; Lindgren & Cousins, 2017). Each of these hypotheses places importance on the amount and configuration of habitat patches and how these determine species persistence. However, these approaches do not take into account the responses of individual animals to habitat fragmentation, which are crucial in determining whether the species is able to persist in fragmented landscapes.

Species‐specific responses can be understood by studying individual movements, which reveal the process by which animals meet their habitat requirements as they respond to heterogeneity in their environment (Johnson, 1980; Jones & Davidson, 2016). Home ranges incorporate all movements of individuals and so provide a useful metric to identify variation in use of space at individual and population levels. There has been a great deal of research on variation in home range size as a function of body size, diet and, more recently, habitat modification (Beasley & Rhodes, 2010; Tucker et al., 2018). For example, larger interpatch distances are expected to hinder reproductive and foraging successes by reducing home range sizes in saw‐whet owls (Hinam & Clair, 2008). In female roe deer, increased edge density provides more foraging opportunities and leads to smaller home ranges (Saïd & Servanty, 2005). Still, there is little information on the extent to which animals can adjust home range area to meet their habitat requirements in fragmented landscapes. Species that can adjust their ranges to incorporate sufficient habitat are less likely to be threatened by fragmentation than species that are restricted to small patches by unsuitability of the surrounding matrix.

Fragmentation is often accompanied by degradation of habitat quality, which can compound habitat loss by rendering patches unusable because they lack the resources to support viable populations (Fischer & Lindenmayer, 2007). Recent studies have found that habitat quality may be more important in shaping the use and viability of habitat in fragmented landscapes (Franken & Hik, 2004; Ye, Skidmore, & Wang, 2013) as seen in butterflies (van Halder, Barnagaud, Jactel, & Barbaro, 2015), pika (Franken & Hik, 2004), and woodpeckers (Robles & Ciudad, 2012). Quality is therefore an important element in understanding how to manage habitat for species.

Here, we investigate how home ranges of a woodland specialist marsupial, the eastern bettong (*Bettongia gaimardi*), are affected by fragmentation of woodland vegetation communities. The eastern bettong is a nonterritorial, small (~1.5 kg) member of the marsupial family Potoroidae. The species is not sexually dimorphic, but does exhibit polygynous mating systems. Females produce 2–3 offspring per year. The species is distributed over the drier eastern half of Tasmania (Rose, 1986) and occurs both in intact and highly fragmented dry sclerophyll woodland. Previous studies estimated home ranges between 32 and 76 ha in intact forest (Taylor, 1993) and showed that distribution was highly influenced by the abundance of ectomycorrhizal fungi (Johnson, 1994; Taylor, 1993). The majority of the remaining wild population of bettongs fall within the fragmented Midlands bioregion on the island state of Tasmania. Occupancy of eastern bettongs in this region is predicted by the quality and amount of habitat within a home range radius (Gardiner, Bain, Hamer, Jones, & Johnson, 2018); however, little is known regarding their movement and response to fragmentation.

Our aim was to determine how fragmentation influences persistence of the eastern bettong, by measuring its influence on individual home ranges. We predicted that if eastern bettongs can compensate for fragmentation through increased movement, their home range size would increase as they expand their range of movement to find essential habitat resources. However, if there are limitations on the ability to move between patches, we expect this would be reflected in a reduction in home range area with increasing fragmentation, as individuals become confined to one or a small number of habitat patches. Habitat degradation is a consequence of the process of habitat fragmentation, both directly and also indirectly as smaller fragments have greater edge effects, in which other impacts such as fire and grazing can affect a higher proportion of the patch. The effects of degradation, however, are frequently considered secondary to fragmentation. There is increasing support for the importance of quality habitat in managing landscapes for biodiversity (Doherty & Driscoll, 2018); therefore, we also tested whether it is as or even more important in influencing home range sizes. Furthermore, we tested the relationship between fragmentation on the amount of habitat used. We predict if individuals cannot compensate for fragmentation, the area of woodland would decrease with increasing fragmentation. However, if the amount of habitat used does not change and or increases with increasing fragmentation then individuals are able to use movement to access the required habitat regardless of fragmentation.

## 2 MATERIALS AND METHOD

### 2.1 Study site

**Figure 1:**
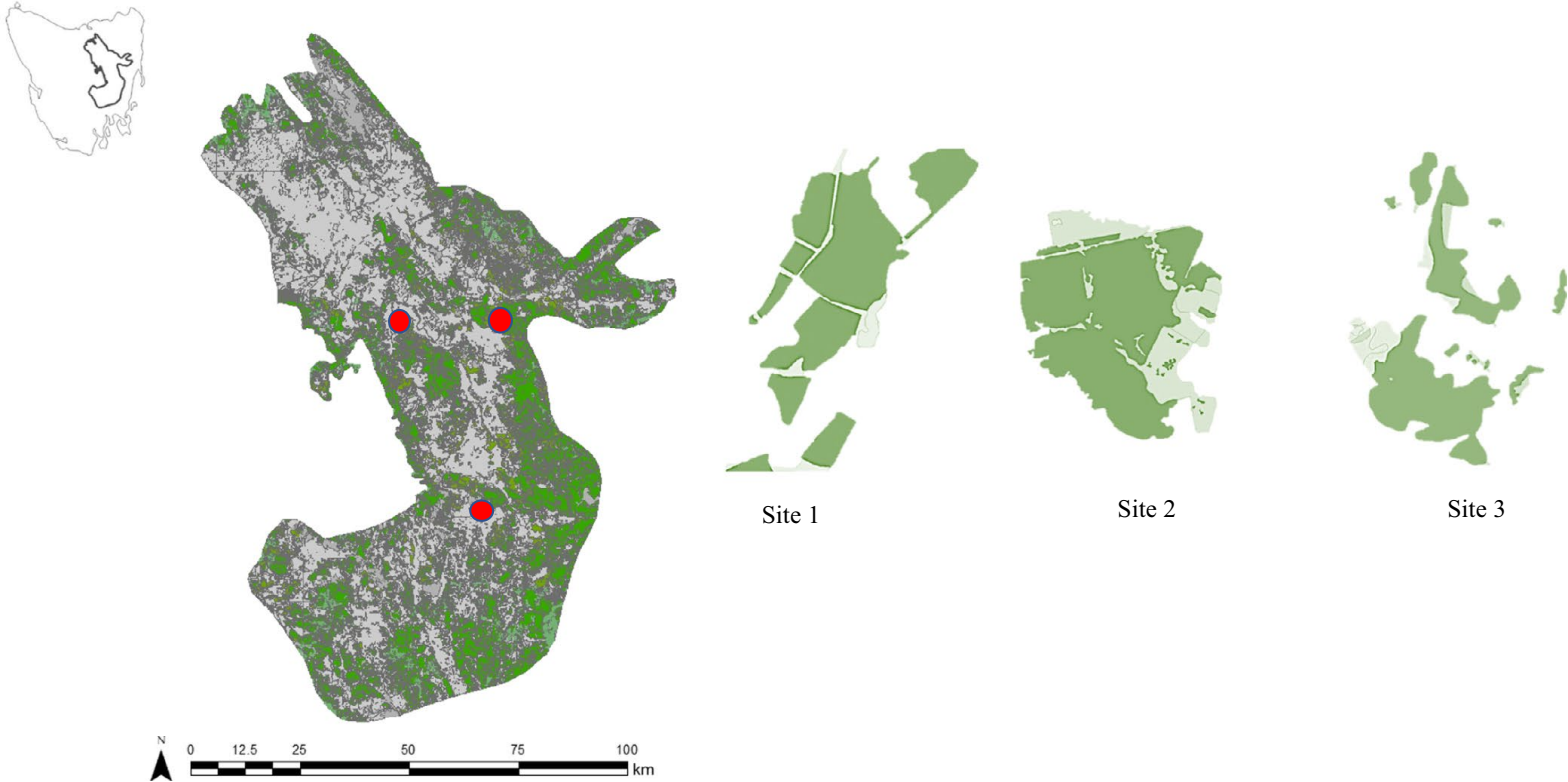
Map showing the Midlands bioregion of Tasmania, Australia, and the location of sites. Red circles represent location of sites where home range estimates were calculated and the corresponding outline of sites; green highlights woodland communities, and gray represents agriculture and urban areas

The Midlands region of Tasmania, Australia, is a national biodiversity hotspot (https://www.environment.gov.au/biodiversity/conservation/hotspots/national-biodiversity-hotspots, accessed 24/05/2018) covering an area of approximately 7,760 km^2^. Before European settlement, the region consisted of grasslands and dry sclerophyll woodlands, but in the last 200 years, the landscape has undergone extensive habitat conversion for livestock production and cropping such that <10% of the original woodland and <3% of original native grassland remains (Jones & Davidson, 2016). The remaining habitat varies in degree of fragmentation and is further threatened by inappropriate fire management and grazing pressure. The Midlands is a dry (500 mm annual average rainfall), cool temperate (annual temperature range −4 to 32°C) region. This study was undertaken at three study areas differing in landscape composition and structure of woodland cover (Figure 1).

### 2.2 Trapping and tracking

Eastern bettongs were trapped between June 2015 and May 2017. Trapping sessions included 3–5 nights of trapping per week for 3 weeks at each site resulting in 305 trap nights. We set 40 wire cage traps (Mascot traps; 30 × 60 cm) before dusk along unsealed tracks at 150 m intervals and baited them with a standard bait of rolled oats mixed with peanut butter and honey. Traps were checked at night, within 5 hr after sunset. Captured animals were permanently identified by subcutaneously inserting PIT tags. Bettongs were weighed and sexed, and individuals weighing more than 1.5 kg were deemed to be adults and were fitted with collars mounted with a GPS logger (G10 UltraLITE GPS logger) and VHF transmitter (Advanced Telemetry Solutions). The GPS units were programmed to take fixes every 15 min between 18:00 and 06:00 hr while animals were active and were retrieved 1 month after deployment. GPS locations collected during the night that an individual was fitted with a collar and during the night the collar was removed were excluded in analyses of home range estimates to prevent bias resulting from trapping and handling disturbance. A total of 26 individual bettongs (14 males, 12 females) across the three fragmented sites were tracked within this study (Table 1).

**Table 1:** Summary of site area and number of individual eastern bettongs in the Midlands bioregion of Tasmania tracked in our study, including their home range size (ha)

Site	Site area (ha)	Tracked individuals	Male Hr range size (ha)	Female Hr size (ha)
1	85	Female = 3 Male = 3	87.19 ± 26.4	58.37 ± 6.52
2	1,291	Female = 4 Male = 4	149.07 ± 31.0	95.80 ± 18.15
3	157	Female = 6 Male = 6	113.1 ± 34.6	79.4 ± 2.87

### 2.3 Home range estimates

We estimated home range sizes from utilization distributions (UD) calculated using Brownian bridge kernels (Horne, Garton, Krone, & Lewis, 2007). This method assumes successive relocations are not independent but instead are time‐dependent. The parameters used include relocations, the distance between relocations, and the Brownian motion variance (the animal's speed between successive locations). This method can generate home range sizes from movement paths, while also including pathways between points, which may be ignored in traditional kernel and MCP analyses (Walter, Fischer, Baruch‐Mordo, & VerCauteren, 2011). Estimates were obtained using the “kernelbb” function in the adehabitatHR and adehabitatLT packages (Calenge & Calenge, 2016) in R version 3.2.1. The UD was calculated for each individual at the 95% isopleths. To remove outliers and potential errors, we chose to use the 95% isopleth in analysis of factors affecting home range sizes. Estimating home range size differences between sexes was analyzed using ANOVAs and standard Tukey Honest significant differences for post hoc comparisons.

### 2.4 Landscape measurements

All locations were overlayed on vegetation maps using TASVEG 3.0 layers provided by TASVEG LIST, which classifies habitat according to the type and community. Where GPS points were present, we classified vegetation into broad communities including woodland, grassland, plantation, and pasture. To gain an understanding of what defines habitat for a species, it is important to measure the elements and characteristics of the local environment that are important to meet the resource needs of the species (Betts et al., 2014; Fahrig, 2013; Johnson, 1980). To ensure each broad community was correctly identified, these were ground truthed while radio‐tracking individuals. Eastern bettongs were found to use (for nesting and foraging) woodland, woodland with grassland understories (several types depending on dominant *Eucalyptus* species), and native plantations. These different vegetation types were combined and classified for the purposes of analysis as habitat.

Previous studies have suggested that stem density of regenerating overstory trees including saplings is an indicator of habitat quality for eastern bettongs (Gardiner et al., 2018). Higher density of stems suggests a larger biomass of fine roots on which mycorrhizal fungi grow, producing the fruit bodies (“truffles”) that are the main food for eastern bettongs. We thus incorporated stem density as a measure of habitat quality. In patches where bettongs were tracked, we placed two 50 m transects, intersecting each other in their middle and running in each cardinal direction. We then recorded the number of regenerating stems of overstory eucalyptus tree species within 5 m either side of the transect. The total counts per patch were calculated as stem density per hectare used as a variable for habitat quality.

### 2.5 Measuring effects of fragmentation on home range sizes

To standardize landscape measurements, we calculated the point of mean activity for each GPS‐tracked animal by calculating the average mean of all recorded locations. First, to test whether bettongs could compensate for fragmentation we placed buffers around each mean center to represent the area that could be used by an individual. A buffer of 250 m radius approximates the core daily active areas within a home range, and the 750 m radius encompasses the total home range area of both sexes.

We used FRAGSTATS (McGarigal, Cushman, & Ene, 2012) to analyze class metrics (defined as the analyses of habitat types) within each buffer size. We measured habitat amount as the proportion of the area within the buffer that was covered by habitat. To quantify fragmentation within the same buffers, we used the CLUMPY index, as a measure of habitat aggregation. CLUMPY is a metric developed to quantify habitat aggregation independently of habitat area (Neel, McGarigal, & Cushman, 2004; Wang, Blanchet, & Koper, 2014). The metric provides a measure which falls between −1 and 1 (i.e., completely disaggregated to completely aggregated).

To determine the best predictors of variation in home range size, we used generalized linear models (GLM) with a log link function in RStudio. Home range size was used as the response variable in all models. Explanatory variables included habitat amount (buffers of 250 and 750 m), habitat aggregation (buffers of 250 and 750 m), and patch habitat quality (Table 2). We expected sex to play a significant role in the variance of home range size and therefore included sex as a parameter in all models.

**Table 2:** Variables used in general linear models to explain home range variations of eastern bettongs in the Midlands bioregion of Tasmania, Australia

Variable	Definition	Min	Max
PW250	Proportion of habitat (woodland, grassland, and native plantation) within a 250 m radius buffer of the mean center	26.3%	100%
PW750	Proportion of habitat (woodland, grassland, and native plantation) within a 750 m radius buffer of the mean center	54.3%	100%
Clumpy250	Metric of aggregation derived from FRAGSTATS within a 250 m radius buffer of the mean center	0.6	1
Clumy750	Metric of aggregation of habitat derived from FRAGSTATS within a 750 m radius buffer of the mean center	−0.1	1
Clumpy_Core	Metric of aggregation derived from FRAGSTATS measured within the estimated core range	−0.6	1
Clumpy_HR	Metric of aggregation derived from FRAGSTATS measured within the estimated home range	0.6	1
Quality	Stem density per hectare of overstory species within habitat patch	0.3	130

Absence of collinearity of our variables was tested using Pearson's correlation and variance inflation factors. Variables with a variance inflation factor of more than 3 suggest severe collinearity (Zuur, Ieno, & Elphick, 2010).

Models were built with single variables as well as all possible combinations of explanatory variables to test our hypotheses across both buffer ranges. Variables measured within a buffer range were only modeled with other variables within the same buffer range; therefore, we ran a total of 10 models. Multi‐model inference (Burnham & Anderson, 2002) was used to determine the models that best described the parameters influencing eastern bettong home range size in response to fragmentation; models were ranked using Akaike's information criterion adjusted for small sample sizes, using the R package MuMIn (Barton, 2009). The final candidate model set included all models within 2 AICc values of the lowest value.

### 2.6 Measuring the relationship between fragmentation and amount of habitat

We further wanted to test the relationship between fragmentation on the amount of habitat used within individual home ranges. We calculated the total area of woodland actually used (defined as Class Area metric in FRAGSTATS); and habitat aggregation (CLUMPY index) in their core range and in their total home range size (see Table 2).

We used generalized linear models (GLM) with a log link function, with amount of woodland as the response variable and habitat aggregation and sex as explanatory variables. We tested a total of five models and ranked models using the method described previously.

## 3 RESULTS

### 3.1 Home range estimates

Home ranges of eastern bettongs overlapped with various vegetation types. On average, these consisted of 80% woodland, 10% plantation, 9% improved pasture, and 1% natural grassland. We observed large variation in home range size among individuals, sexes, and sites (Table 1). Males had larger home ranges than females (*F*_1, 2_ = 4.54, *p* = .04). Mean home range size was 118 ha (*SD*: 39.2; range 87–149 ha) in males, and 80 ha (*SD*: 20.3; range 58–95 ha) in females (Table 1).

### 3.2 Measuring the effects of fragmentation on home range sizes

**Figure 2:**
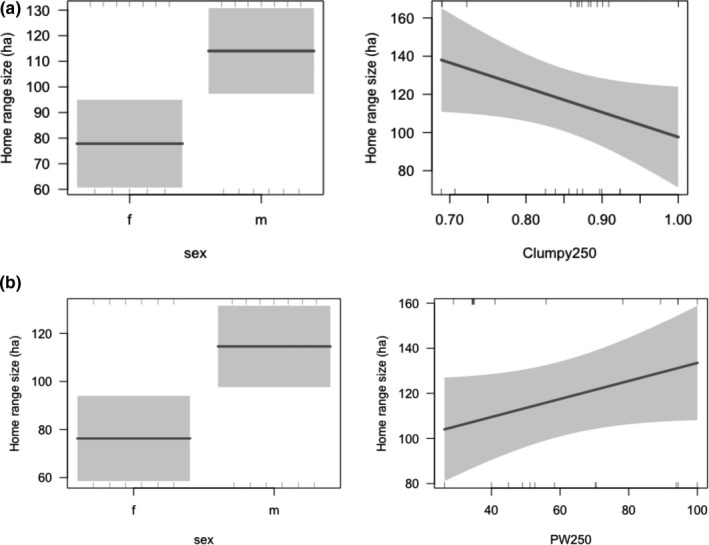
Top model estimates showing the partial residuals of (a) sex and habitat aggregation within a core range buffer (CLUMPY250) and (b) sex and proportion of habitat in the core range buffer (PW250), as well as 95% confidence intervals

There were two models predicting home range size that were within 2 AICc values of each other (Table 3). The top model, with an AICc weight of 0.37, included sex and the aggregation index of habitat within a radius of 250 m of the range center (representing daily activity; Table 3). Males had larger home ranges than females, and ranges were larger when the habitat was more disaggregated (Figure 2). The second model, with an AICc weight of 0.25, included sex and the amount of habitat within 250 m of the range center (representing daily activity) range. Males had larger home ranges than females and home range size increased with the amount of habitat available. There was little support for models including the aggregation index and amount of habitat at the larger buffer size. These did not appear in any models in the final set suggesting habitat configuration and amount at the core range is more important in determining home range size variations in fragmented landscapes.

**Table 3:** Candidate models used to determine the parameters that influence home range size including the sex of the individual, the quality of the patch (stem density of the patch used by individuals), the amount of habitat (PW), and the aggregation of habitat (Clumpy) measured within buffers representing the core range size (250 m) and the home range size (750 m)

Model	*K*	AICc	dAICc	AICc W
Sex+Clumpy250	4	247.93	0	0.37
Sex+PW250	4	248.69	0.76	0.25
Sex+Quality+Clumpy250	5	250.93	3	0.08
Sex+Quality	4	251.15	3.22	0.07
Sex+PW750	4	251.56	3.63	0.06
Sex+Clumpy750	4	251.58	3.65	0.06
Sex+Quality+PW250	5	251.82	3.89	0.05
Sex+Quality+PW750	5	254.28	6.35	0.02
Sex+Quality+Clumpy750	5	254.28	6.36	0.02
Null	2	254.31	6.38	0.02

### 3.3 Measuring the effects of fragmentation on amount of woodland

**Figure 3:**
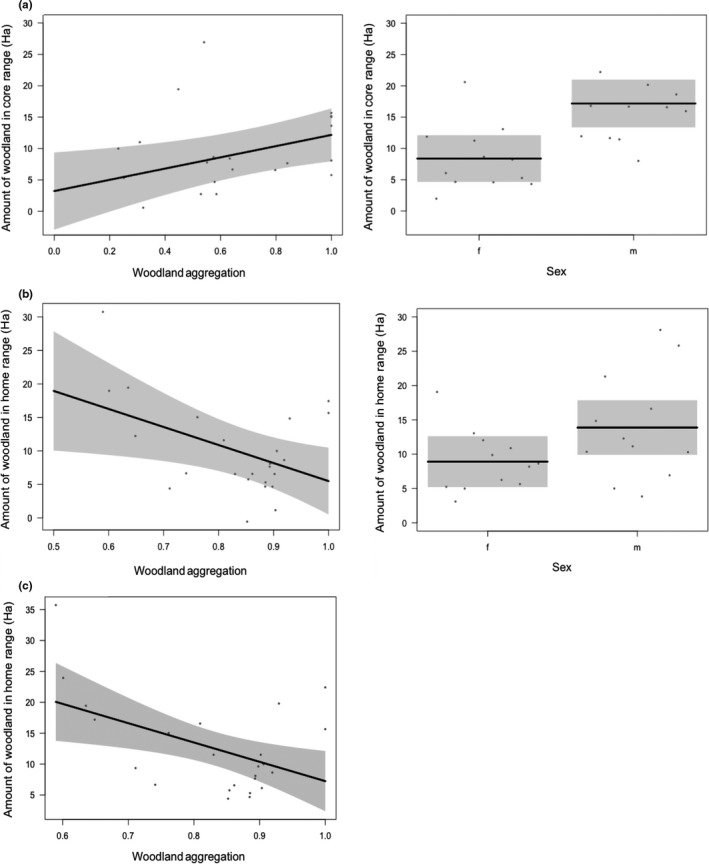
Top model estimates showing partial residuals and 95% CI, testing the relationship of fragmentation on the amount of woodland within (a) a bettong's estimated core range and sex; (b) a bettong's estimated home range and sex; and (c) a bettong's estimate home range

There were three models within 2 delta AICc of each other (Table 4). The first included habitat aggregation measured at the core range scale and sex of the individual, with an AICc weight of 0.43. The model suggests that at the core range, the amount of habitat used increases with increasing habitat aggregation. The second model included habitat aggregation measured at the home range size and sex of the individual with an AICc weight of 0.3. At the home range scale, the amount of habitat used increases when habitat is more disaggregated (Figure 3). The third model only included aggregation at the home range scale with similar results to the second model, but with an AICc weight of 0.2

**Table 4:** Candidate models used to determine the relationship between fragmentation and the amount of woodland used in a bettong's core and home range size

Model	*K*	AICc	dAICc	AICc W
Clumpy_core+sex	4	164.16	0	0.43
Clumpy_HR+sex	4	164.86	0.69	0.3
Clumpy_HR	3	165.52	1.35	0.22
Null	2	169.27	5.1	0.03
Clumpy_core	3	170.41	6.24	0.02

## 4 DISCUSSION

We tested species response to fragmentation using a woodland specialist, the eastern bettong, in an agricultural landscape as a study system, investigating how the amount, configuration, and quality of habitat patches influence movement ranges, which is the process by which animals respond to landscape. Our results suggest that the sex of the individual, the degree of aggregation of woodland communities, and the amount of woodland within the daily movement range influence home range size. As woodland patches become more disaggregated, bettong home range size increases, probably because they need to cross gaps (pasture) to reach sufficient woodland to fulfill their food requirements. Where there are more patches available within traveling distance, home range size increases because bettongs are crossing more gaps and covering a greater total area. Overall, our results support the hypothesis that individuals can compensate for fragmentation by increasing their ranges depending on the spatial scale of interest.

The scale at which landscape structure is measured is important for species persistence and can facilitate at what scales management should focus their efforts. Previous studies have suggested that the “scale effect” should vary as each spatial scale is linked to different important ecological processes (Gestich, Arroyo‐Rodríguez, Ribeiro, Cunha, & Setz, 2019; Jackson & Fahrig, 2012; Miguet, Jackson, Jackson, Martin, & Fahrig, 2016) and therefore is species dependent. For example, at the core range, resource acquisition and availability are important for individual survival and breeding outcomes. At larger spatial scales, processes such as dispersal are more important for population viability and genetic diversity. In this study, we show support for scale effect being dependent on species' mobility, a result similarly by Jackson and Fahrig (2012). Our results suggest that as fragmentation increases, bettongs decrease their core range size, but increase their overall home range size given the amount of habitat and fragmentation at each spatial scale. At present, our results suggest bettongs can compensate for fragmentation given they have enough habitat within a home range size. Indeed, if the amount of habitat is too small at the core range size, it is likely individual survival and population establishment would be low as resource abundance decreases. Incorporating various spatial scales to determine how animals respond to habitat fragmentation is still relatively new. Including a range of spatial scales in future studies could be important toward understanding the effect of landscape structure on other movement‐dependent processes across life history stages such as dispersal and genetic connectivity.

Requirements for habitat area are species‐specific, reflecting the particular resource needs and movement capacity of individual species. As a dietary specialist on hypogeal fungi that grow in eucalypt woodland communities, the eastern bettong has a relatively large home range for its body size. We show that individuals can vary their core range area in response to the amount and configuration of woodland vegetation in a fragmented agricultural landscape. Eastern bettongs can probably do this because they are able to rapidly cross gaps to cover the amount of habitat area they need to meet their requirements and also reach other woodland patches. This is congruent with the observation that species that have higher mobility are able to accommodate their habitat area requirements via movement (Anderson et al., 2005; Martin & Fahrig, 2016; Saïd & Servanty, 2005). In this case, patch configuration and habitat amount within the core range do not necessarily restrict habitat use; rather, small and isolated patches can be of value because they contribute to the total habitat amount that each individual requires for a viable home range. The ability to compensate is advantageous in fragmented landscapes, increasing opportunities to exploit multiple patches and their resources, therefore influencing survival.

Our findings highlighted the importance of the individual's sex in habitat use and home range size. As reported across many species and studies, these differences are fundamentally linked to each sex resource requirement. For males, this is usually driven by maximizing breeding opportunities, while for females, this often includes food and shelter for themselves and offspring (Sunde, Redpath, & Kelt, 2006). Given the significantly smaller ranges of females to males within our study, they are likely to be more impacted by further habitat loss and fragmentation at the core range size (Arroyo‐Rodríguez, González‐Perez, Garmendia, Solà, & Estrada, 2013; Smith & Hellmann, 2002). This can have negative implications on population demographics, population recruitment, and therefore persistence. This further highlights the importance of incorporating various spatial scales to ensure essential habitat structure is maintained for all individuals. Future studies could further understand the impacts of fragmentation on demographics by highlighting differences in daily movement between the sexes and determine the impacts of fragmentation across life stages, for example with and without offspring.

Our results support thinking that habitat fragmentation may not be negative per se (Fahrig et al., 2019 but see Fletcher et al., 2018). We show that individual bettongs can increase their ranges when habitat is more disaggregated and when habitat amount increases. By removing the strict delineation of patches and testing the effects at the landscape scale, the habitat amount hypothesis values smaller patches, which are often disregarded or considered unusable. Small patches can contribute to the total amount of habitat available which can be valuable (Tulloch, Barnes, Ringma, Fuller, & Watson, 2016) for movement (Barbosa, Knogge, Develey, Jenkins, & Uezu, 2017), resting, and refuge (Machado, Fontes, Santos, Garcia, & Farrapo, 2016). This suggests that mobile species can cope with a certain degree of fragmentation if they can access enough habitat within their range. Of course, this applies to areas where there is sufficient habitat for populations to persist. Our models suggest that landscape characteristics at the core range are the most important—in cases where there is not sufficient habitat to establish a core range it is likely that populations would not be able to persist. Therefore, increasing habitat amount is a beneficial conservation strategy; adding native vegetation as corridors or to smaller patches to promote connectivity may improve species movement and decrease the amount of time spent searching for habitat, and exposed to edges or the matrix.

## CONFLICT OF INTEREST

None declared.

## AUTHOR CONTRIBUTION

RG, MJ, and CJ conceived the ideas and designed the methodology; RG and KP collected the data; RG and SC analyzed the data; RG, MJ, and CJ led the writing of the manuscript. All authors contributed to the final manuscript for publication.

## Data Availability

https://doi.org/10.5061/dryad.cjsxksn22
